# Development of a New Integrated System for Vital Sign Monitoring in Small Animals

**DOI:** 10.3390/s22114264

**Published:** 2022-06-03

**Authors:** Regina G. Oliveira, Pedro M. M. Correia, Ana L. M. Silva, Pedro M. C. C. Encarnação, Fabiana M. Ribeiro, Ismael F. Castro, João F. C. A. Veloso

**Affiliations:** 1 Institute for Nanostructures, Nanomodelling and Nanofabrication (i3N), Physics Department, University of Aveiro, 3810-193 Aveiro, Portugal; regina.oliveira@ua.pt (R.G.O.); analuisa.silva@ua.pt (A.L.M.S.); pedro.encarnacao@ua.pt (P.M.C.C.E.); fabiana.ribeiro@ua.pt (F.M.R.); joao.veloso@ua.pt (J.F.C.A.V.); 2 Radiation Imaging Technologies Lda. (RI-TE), University of Aveiro Incubator, PCI—Creative Science Park, 3830-352 Ílhavo, Portugal; filipe.castro@ri-te.pt

**Keywords:** body temperature, heart rate, Positron Emission Tomography (PET), respiratory rate, small animal imaging, vital sign monitoring

## Abstract

Monitoring the vital signs of mice is an essential practice during imaging procedures to avoid populational losses and improve image quality. For this purpose, a system based on a set of devices (piezoelectric sensor, optical module and thermistor) able to detect the heart rate, respiratory rate, body temperature and arterial blood oxygen saturation ($SpO_{2}$) in mice anesthetized with sevoflurane was implemented. Results were validated by comparison with the reported literature on similar anesthetics. A new non-invasive electrocardiogram (ECG) module was developed, and its first results reflect the viability of its integration in the system. The sensors were strategically positioned on mice, and the signals were acquired through a custom-made printed circuit board during imaging procedures with a micro-PET (Positron Emission Tomography). For sevoflurane concentration of 1.5%, the average values obtained were: 388 bpm (beats/minute), 124 rpm (respirations/minute) and 88.9% for the heart rate, respiratory rate and $SpO_{2}$, respectively. From the ECG information, the value obtained for the heart rate was around 352 bpm for injectable anesthesia. The results compare favorably to the ones established in the literature, proving the reliability of the proposed system. The ECG measurements show its potential for mice heart monitoring during imaging acquisitions and thus for integration into the developed system.

## 1. Introduction

Imaging techniques can be used during preclinical research to analyze what is happening at anatomical and/or functional levels in animal models such as mice. The quality of the resulting image is strongly affected by the animal movements during the exam, even the ones resultant from physiological systems, such as breathing and heart movements during the respiratory and cardiac cycles, respectively. In order to diminish the influence of these motions, some imaging systems operate using gating techniques, where images are acquired at specific periods of the respiratory and cardiac cycles [1,2]. Moreover, the animals are usually restrained and anesthetized to reduce the non-physiological motions. In general, anesthetics affect mice’s autonomic nervous system, leading to changes in some of their vital parameters, such as respiratory frequency, heart rate and body temperature [1,3,4].

Thus, it is mandatory to monitor the vital signs during imaging procedures not only to help to implement corrections during the image reconstruction phase or in the gating techniques, but also to guarantee the animal’s well-being during these procedures, avoiding population losses, and in some way, controling the depth of anesthesia, thus preventing the mouse from waking up before the end of the experiment [4,5]. This control is in accordance with the Refinement principle of the 3R’s directive (Replacement, Reduction, and Refinement), which intends to minimize the animal’s stress and pain and improve as possible its welfare during its life and in these kinds of imaging experiments. In addition, the use of non-invasive sensors to measure the animal’s vital signs refines the procedures, since these devices cause less stress to the animal [6].

Vital sign monitoring systems available in the market and that are suitable for preclinical imaging purposes are mostly developed by scanner manufacturers and consequently are designed for their imaging systems, providing little functional information. There are other monitoring systems in the market for the monitoring of small animals during surgeries or other experimental procedures, but they are costly, bulky and not adaptable to the imaging system size. In this case, the researchers have to design and customize their own systems, such as those developed by Meleppat et al. [7] for animal heating and temperature monitoring and those by Muller et al. [8] for respiratory monitoring, body temperature measurement and bed heating.

In this work, a system (hardware and software) able to record simultaneously signals from different non-invasive devices—optical module, piezoelectric sensor and thermistor—is proposed. With the optical module, it was possible to record the heart and respiratory rates as well as the arterial blood oxygen saturation ($SpO_{2}$). The heart and breathing signals were also detected with the piezoelectric sensor. The thermistor was used to access the mice’s body temperature. Moreover, a dedicated non-invasive electrocardiogram (ECG) module was used to record the heart’s electrophysiological activity and evaluate the possibility of its integration with the other sensors. Furthermore, the characterization of these devices with a fully operational imaging system was performed. The designed system is being developed to be a universal animal monitoring and positioning device in the preclinical imaging field and to be used in a wide range of imaging scenarios without significant modifications.

## 2. Materials and Methods

### 2.1. Imaging System—easyPET.3D

**Figure 1:**
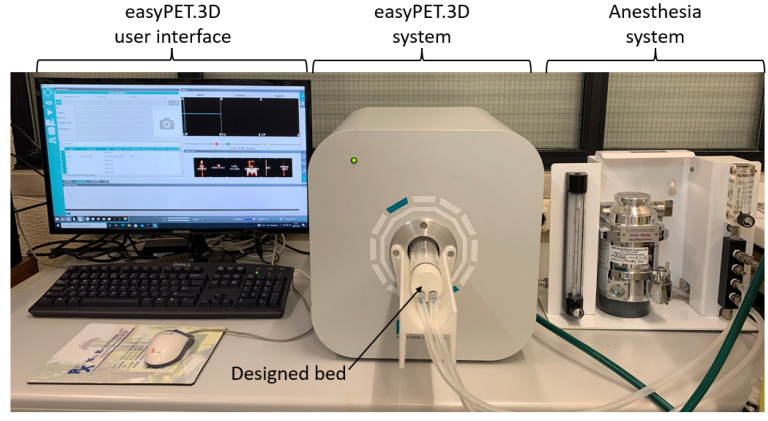
easyPET.3D benchtop system with the evidence of its user interface and the mouse bed. The anesthesia system and its connection to the mouse bed are also observable in this figure.

The easyPET.3D is a micro-Positron Emission Tomography (PET) system developed at Aveiro University that makes use of only two pairs of detector blocks (2 blocks with 32 detector cells each), arranged in a U-shaped PCB (Printed Circuit Board). The board rotates around the center, where the mouse is positioned, covering the same field of view as the conventional micro-PET systems. The easyPET.3D distinguishes itself from the other systems by its performance, cost and simplicity [9]. In Figure 1 is presented the easyPET.3D imaging system, as well as its user interface and mouse bed. The gaseous anesthesia system used in this work is also evidenced in Figure 1.

### 2.2. easyPET.3d Mouse Bed

**Figure 2:**
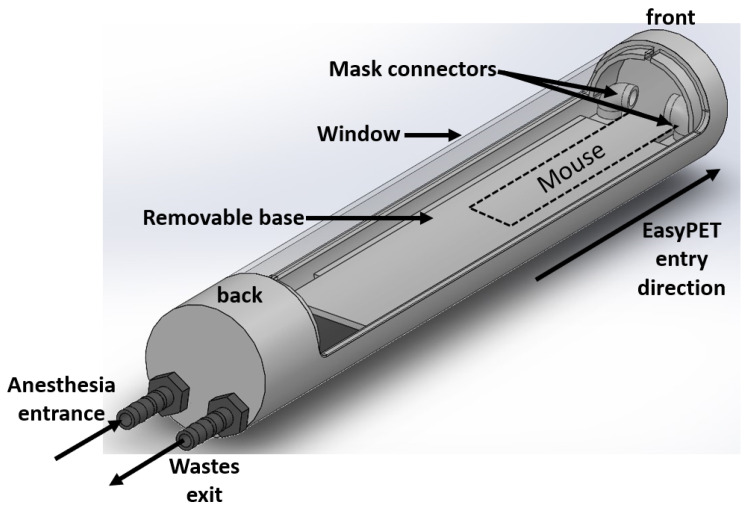
easyPET.3D bed prototype, with the representations of its several components.

A bed is an essential piece of any imaging system, with the main purpose of adequate animal positioning and restraining during the exam. A functional and dedicated bed prototype was designed for the easyPET.3D system, which is represented in Figure 2. This bed has a cylindrical shape, with 40 mm diameter and 260 mm length, manufactured by a 3D printer. The window is removable and transparent, which allows easy access and manipulation of the mouse during the procedure, as well as observation of the animal. This prototype can be connected to an external gaseous anesthesia system, since it has an in/out air passage system allowing the gas to flow through it. The mask connectors join the air flowing system to a breathing mask, where the mouse’s nose is placed for efficient and controlled anesthesia. All the bed structure and components are printed in a plastic material to reduce the radiation attenuation.

The sensors are strategically positioned on the bed’s base to record the vital data. Under the removable base (see Figure 2), a required free space has been considered for future implementations, e.g., heating system.

### 2.3. Animal Model—Mice

The mice used during this work were BALB/c and BALB/c nude species. They were employed in other current studies at iCBR (Institute for Clinical and Biomedical Research, Coimbra, Portugal), and their vital signs were recorded during the PET exam with the piezoelectric sensor, optical module and thermistor. The ECG was acquired before the PET acquisition. This work was carried out with the authorization and under the guidelines of ORBEA—*Órgão Responsável pelo Bem-Estar dos Animais* (Organ Responsible for Animal Welfare) 17/2015 and DGAV—*Direção-Geral da Alimentação e Veterinária* (General Directorate of Food and Veterinary Medicine).

### 2.4. Anesthesia Protocols

During the imaging procedures, sevoflurane was used as an inhalatory anesthetic agent. Its effects on animals’ physiological systems are very similar to the ones produced by isoflurane, which is very well documented for mice [10]. The anesthesia was introduced to the mouse in a separate chamber, which was flooded with a gas mixture of air with 5% of sevoflurane. As soon as the mouse stood still and did not respond to external stimuli, it was transferred to the bed with the integrated anesthesia gas flow system, where the anesthesia was reduced to 1.5–2.5% of sevoflurane in air. Throughout the tests, the anesthesia concentration was smoothly adjusted to keep the mouse stable and to observe the sevoflurane influence on the cardiovascular and respiratory systems. In the case of the non-invasive ECG system, the mice were anesthetized with a mixture of ketamine and chlorpromazine (0.2 mL) injected via intraperitoneal, with the following formulation: ketamine (1.5 mg/mg weight) + chlorpromazine (0.05 mg/mg weight) (3:1): saline (1:1). This anesthesia method was used due to the unavailability of the gas anesthesia system during these studies.

### 2.5. Vital Sign Measuring Devices

**Figure 3:**
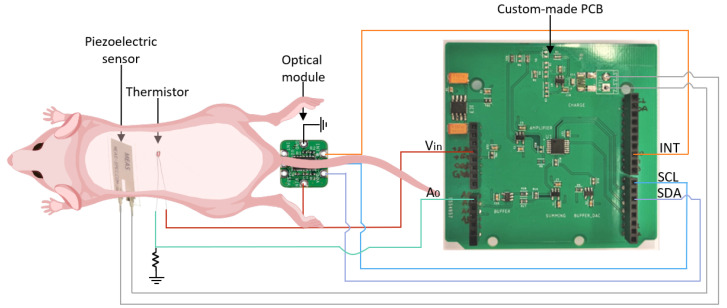
Schematic of BALB/c nude mouse positioning in the system and the sensor’s connections to the custom-made PCB. The piezoelectric sensor is under the mouse’s thoracic cage, the thermistor is under the belly, and the optical module is positioned below the tail’s basis. Vin—input voltage for the optical module and thermistor; A${}_{0}$—ADC channel for the thermistor data; INT—Active-Low Interrupt (Open-Drain) of the optical module; SCL—optical module I2C clock input; SDA—optical module I2C data.

**Figure 4:**
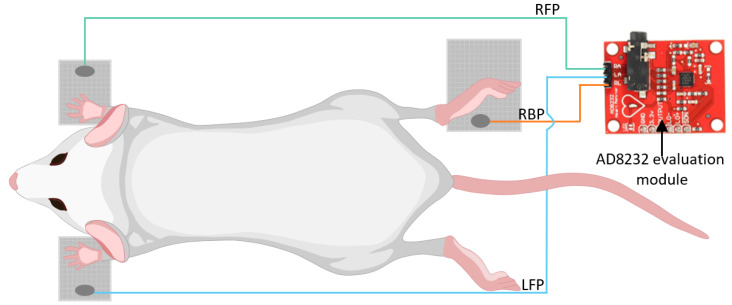
BALB/c mouse positioning in the ECG system with the 3 dry electrodes configuration and connections to the AD8232 evaluation module. RBP—Right Back Paw; RFP—Right Front Paw; LFP—Left Front Paw.

The non-invasive devices used in this work to measure the heart rate, respiratory rate, $SpO_{2}$ and body temperature were an optical module, a piezoelectric sensor and a thermistor. The sensor’s low volume, mass and dimensions were the main reasons to choose it, notably to reduce disturbance on image formation and to be suitable for the mouse’s body size. The sensor’s positioning under the mouse can be observed in Figure 3. Furthermore, the non-invasive ECG system is presented and described in Figure 4.

#### 2.5.1. Optical Module

MAXREFDES117 is an optical module designed to monitor the heart rate and $SpO_{2}$ in reflective mode [11]. The main component of this module is the MAX30102, which is essentially composed of two LEDs (Light-Emitting Diodes)—one in the red zone (660 nm) and another one in the infrared (IR) zone (880 nm)—and a photoreceptor [12].

This module was able to monitor the mouse’s heart rate, respiratory rate and $SpO_{2}$, having an adequate size for this purpose. It was positioned under the mouse tail’s base (see Figure 3), making this system suitable for BALB/c and BALB/c nude mice.

#### 2.5.2. Piezoelectric Sensor

The piezoelectric sensor used in this work was the LDT0-28k from Measurement Specialties, with a piezoelectric polymeric film in its composition. This sensor is recommended for vibration sensing and body movement detection [13]. The LDT0-28k was used to monitor the mice’s heart and respiratory rates. It was placed under the thoracic cage since it is where the signals under study have higher amplitude.

#### 2.5.3. Thermistor

The sensor applied to measure the mice’s body temperature was a thermistor. More specifically, an NTC (Negative Temperature Coefficient) thermistor was used, with a resistance of 100 kΩ, at room temperature [14]. The thermistor was put under the belly to ensure the sensor coverage.

#### 2.5.4. Non-Invasive ECG

A non-invasive ECG with a 3-electrode geometry (2 positioned under the front paws and 1 under the right back paw) was used and tested to measure the heart rate; it is represented in Figure 4. In order to acquire the signal from the electrodes, the AD8232 front-end module was chosen, since it has the necessary electronics implemented and it allows the use of customized electrodes [15]. In this work, dry electrodes were employed, made of a sheet of Cu/Ni polyester film electrically connected to the AD8232 [16].

### 2.6. Signal Acquisition

#### 2.6.1. Hardware

To acquire the signals from the piezoelectric sensor, optical module and thermistor simultaneously, a readout board was designed. The PCB was dedicated to the piezoelectric sensor since the datasheet from this sensor recommends the use of a charge amplifier. The resultant PCB for this sensor can be seen in Figure 3, where the other sensors were connected to the lateral black pins of the commercial board Nucleo-F446RE, built with a STM32F446RE, an ARM 32-bit microcontroller.

**Figure 5:**
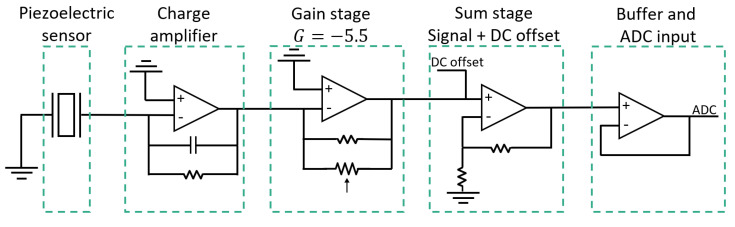
Schematic of the designed circuit for the piezoelectric sensor, with the signal modulation stages and optimized value for gain (G).

The designed circuit for this board includes some stages for the readout of the signal from the piezoelectric sensor, which are represented in Figure 5 and explained as follows:

- The first stage is a charge amplifier responsible for converting the charge generated from the piezoelectric material into a usable voltage, which can be further amplified in the next stages [17];
- Following the charge amplifier, a gain stage was implemented with an inverting amplifier setup, where the gain value is adjustable by firmware using a digital potentiometer;
- The next stage is necessary to control the offset voltage of the signal by summing an external voltage defined by software to the signal from the previous stage, ensuring that it is between 0 and 5 V, which is the microcontroller ADC’s (Analog to Digital Converter) input range;
- Before entering the assigned ADC (A1), the signal passes through a buffer stage, which truncates the signal between 0 and 5 V, to be readable by this port.

#### 2.6.2. Software

Besides the necessary hardware, it was also needed to develop firmware to simultaneously control the sensor’s acquired data and send them to the computer to be processed.

**Figure 6:**
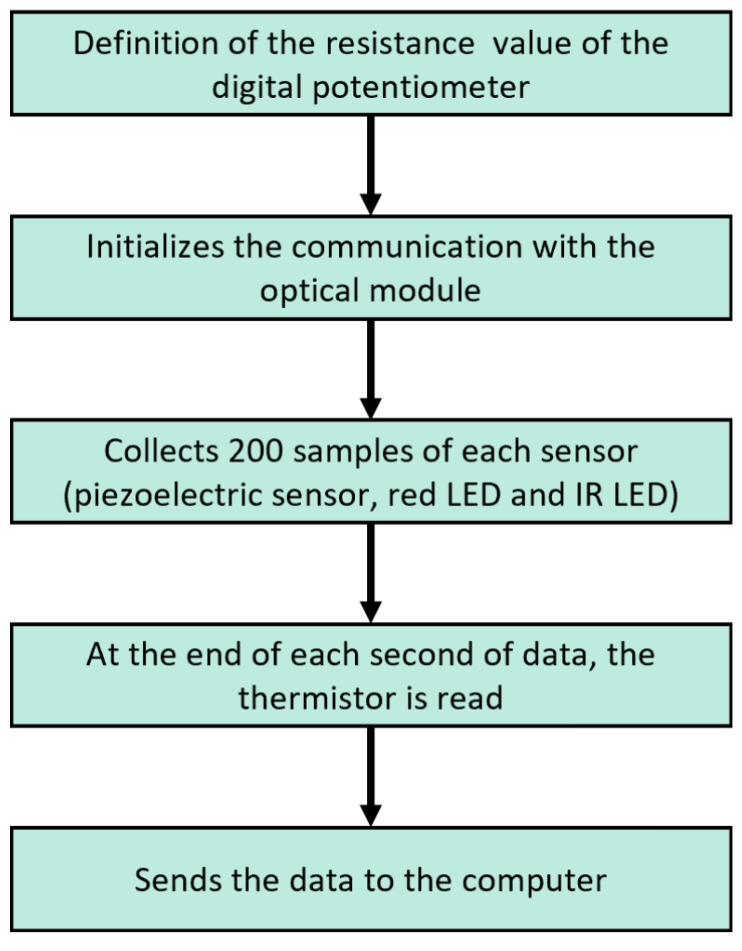
Firmware schematic.

The firmware running in the microcontroller was developed using the Mbed Online Compiler, and its features are summarized in Figure 6. The data from the optical module, piezoelectric sensor and thermistor are acquired at 200 Hz, allowing the system to detect the mouse’s heart rate, which can achieve 600 bpm (beats per minute), even under anesthesia [18].

**Figure 7:**
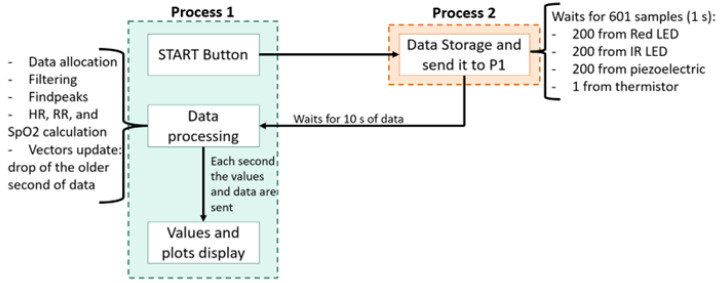
Schematic of the software stages. The software is divided into processes (1 and 2), which work in parallel to allow real-time acquisition and processing. HR—Heart Rate; RR—Respiratory Rate.

To process the data received by the computer, software was developed using Python 3.9 [19]. The created software is composed of two processes (Process 1 and Process 2) running in parallel computer processor cores. The software’s workflow is represented in Figure 7. Firstly, the data were saved in files and then, the data from each sensor were filtered with a Butterworth bandpass filter. The cut-off frequencies of the filter, using sevoflurane anesthesia, were 0.5 Hz and 3.5 Hz to detect the respiratory wave and 5.5 Hz and 8 Hz to detect the heartbeat wave. After this filtering stage, the wave peaks were found using the Python’s findpeaks algorithm, and the respiratory rate and heart rate from each sensor were calculated [20]. These values, as well as the body temperature, the real-time heartbeat plot and the respiratory wave, were displayed in a graphical user interface (GUI), which also controls the beginning and the end of acquisition.

For the non-invasive ECG, the process of data acquisition was very similar to the other sensors. However, the filtering cut-off frequencies were 1 Hz and 20 Hz for the filter’s lower and higher edge, respectively. These bandpass frequencies were chosen to detect all the features inherent to the ECG wave. The heart rate was computed by detecting the wave’s R peaks.

## 3. Analysis and Discussion of Results

**Figure 8:**
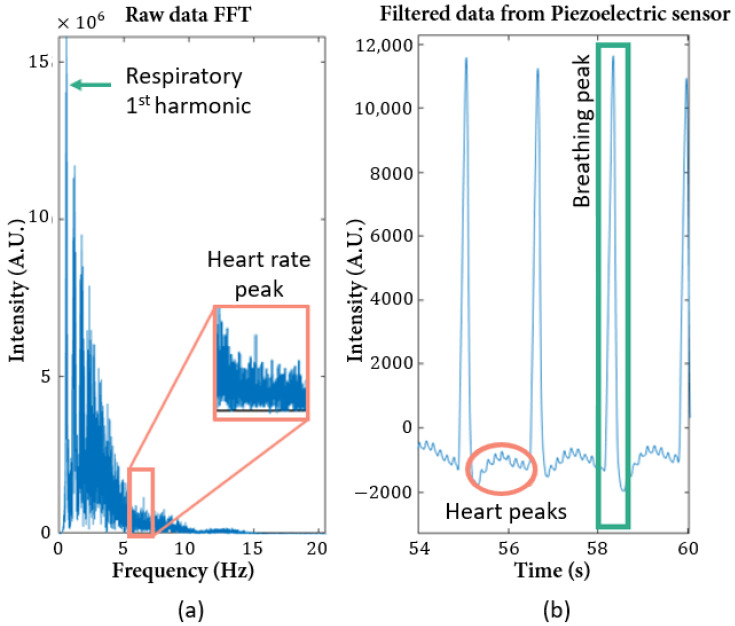
Piezoelectric signal analysis, with 5% of sevoflurane (Mouse 2): (**a**) representation of the absolute value of the signal’s FFT; (**b**) signal filtered with a bandpass filter of 0.1 and 15 Hz as cut-off frequencies, where it is possible to clearly distinguish the heartbeat wave from the respiratory wave.

The first approach to analyze the signal was to plot the signal’s Fast Fourier Transform (FFT) absolute value, to understand the frequencies that are present. The respiratory harmonics are very visible in this graphic (Figure 8a), and the first one corresponds to the expected value for the considered anesthesia concentration. However, the peak that appears in the predicted interval for the heart rate (5–8 Hz) is almost imperceptible between noisy frequencies. Thus, it was necessary to analyze the signal after filtration with a large bandpass filter, which is represented in Figure 8b, and to count the peaks between two consecutive respiratory peaks, to confirm the frequency range for the heart rate values.

### 3.1. Respiratory Rate

**Figure 9:**
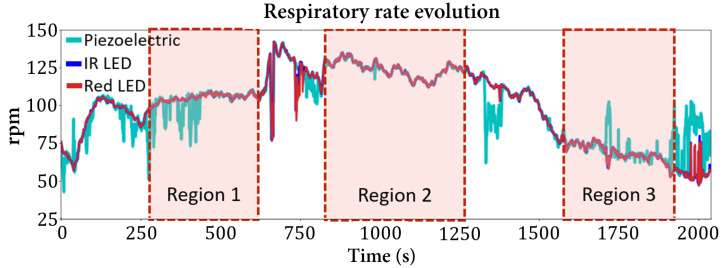
Respiratory rate evolution (Mouse 1) through sevoflurane concentrations changes. The highlighted regions represent moments where the anesthetic concentration did not change: Region 1—stable zone at 2% of sevoflurane; Region 2—stable zone at 1.5% of sevoflurane; Region 3—stable zone at 4% of sevoflurane.

The respiratory rate evolution over time for Mouse 1 is presented in Figure 9, where three regions with a stable concentration of sevoflurane are highlighted. The values from these regions are used to calculate a mean value of the respiratory rate and the corresponding uncertainty, which are presented in Table 1. Between these regions, the concentration of sevoflurane was changed, representing transition zones between the established concentrations for signal acquisition.

The influence of the anesthetic agent concentration on the respiratory rate is evidenced in Figure 9 and in the obtained values presented in Table 1, from which a correlation is found: higher doses of anesthetic lead to lower values of respiratory rate and vice versa, which are in accordance with the reported literature [21,22].

For sevoflurane at 1.5%, the obtained value of 124 rpm (respirations per minute) is very close to the one reported for isoflurane anesthesia (137–151 rpm [23]). The breathing rate is strongly affected by the anesthesia type and concentration, as reported in [22]. The calculated value is not within the expected range, which can be explained by the use of another anesthetic agent that can induce this small difference, as well as by the manual adjustment of the anesthesia concentration, instead of a digital control as in [23], leading to a lower control of the actual concentration, possibly affecting the respiratory rate.

### 3.2. Heart Rate

**Figure 10:**
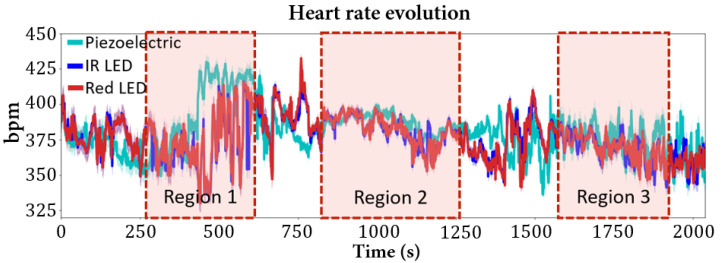
Heart rate evolution (Mouse 1) through sevoflurane concentration changes. The highlighted regions represent moments where the anesthetic concentration did not change: Region 1—stable zone at 2% of sevoflurane; Region 2—stable zone at 1.5% of sevoflurane; Region 3—stable zone at 4% of sevoflurane.

The heart rate did not vary as much as the respiratory rate with the variation of sevoflurane concentration, as can be seen in the values obtained from the several sensors in Table 2 and in the follow-up of the values’ evolution over time in Figure 10. This behavior is reported in the literature for gaseous anesthesia, which conserves the cardiovascular function very well [1,22].

The graphic represented in Figure 10 is related to Mouse 1, and it is possible to see that the values are between 350–400 bpm. The existing fluctuations are due to mouse movements and periods with lower signal quality.

For sevoflurane at 1.5%, the obtained heart rate mean value was 387 bpm for the piezoelectric sensor and 388 for the IR and Red LEDs. The value range of this sevoflurane concentration region is within the reported limits for isoflurane (391–425 bpm [23]). Despite the minor differences, which can be related with experimental conditions or with the reduced number of studied animals needed to provide statistical significance, it is possible to affirm that the designed system can detect the heart rate consistently.

### 3.3. $SpO_{2}$

$SpO_{2}$ values for this anesthesia were not found in the literature for BALB/c mice. However, some studies with other strains were made, such as the one from Tsukamoto et al., which employed ddY mice anesthetized with isoflurane at 2%, obtaining $SpO_{2}$ values above 90% [3]. The results obtained with the designed system, presented in Table 3, were close to the reported values and can be considered consistent because of the strain variance. Furthermore, when the respiratory cycle is suppressed, the $CO_{2}$ levels increase, which leads to lower blood pH, consequently decreasing the hemoglobin oxygen uptake, even if the mice are provided with oxygen, which is the case, therefore explaining these $SpO_{2}$ values [21,24].

### 3.4. Body Temperature

The temperature interval for BALB/c mice measured with the external thermistor is presented in Table 4, revealing a higher temperature limit for each mouse that turned out to be lower than expected. Since the thermistor was not in direct contact with the mouse’s skin, the body temperature was not measured with the required accuracy, which could explain this temperature undervaluation.

**Figure 11:**
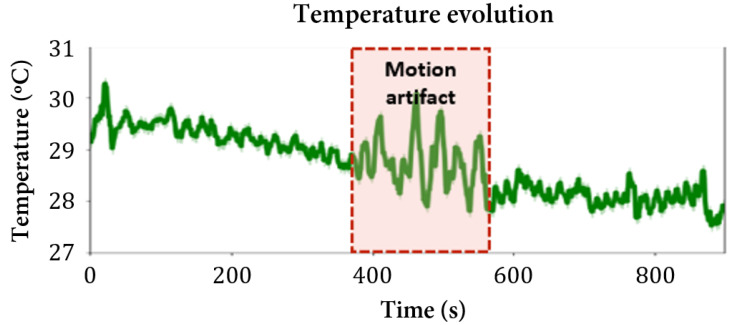
Mouse 2 temperature evolution. Of note is the downward tendency of temperature over time and the influence of motion artifacts in the measurement.

However, as can be seen in Figure 11, the thermistor proved to be able to detect the decrease in the temperature values over time, which can be sufficient to detect hypothermia, a common secondary effect of anesthesia. The temperature evolves from higher values to lower ones, as expected since the mice did not have an available heat source to preserve their temperature in a sharp interval.

### 3.5. Non-Invasive ECG

**Figure 12:**
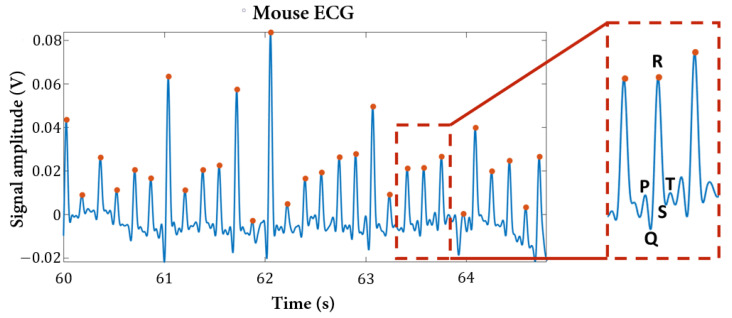
ECG signal from the non-invasive system with dry electrodes. The observed signal was filtered with a bandpass filter between 1 and 20 Hz, to be possible to capture all the ECG waves (P, QRS complex and T) highlighted in the rectangle.

The non-invasive ECG system was tested with BALB/c nude mice anesthetized with ketamine and chlorpromazine, which was the only anesthesia available at the moment of the experiment. Although some mice movements were observed, from one of the gathered ECG segments after filtering, highlighted in Figure 12, it is possible to observe the characteristic features of this wave, the P wave, the QRS complex and the T wave. The obtained heart rate value was calculated from the difference of consecutive QRS complexes (identified by the dots in Figure 12), and for this segment and anesthesia, the computed value was 352 ± 12 bpm. The presented results for this ECG system show the reliability of its implementation with the other sensors, allowing the user to access the heart’s electrophysiological information.

## 4. Conclusions

These results have shown the feasibility of the proposed integrated system to successfully detect the heartbeat wave, the respiratory wave, $SpO_{2}$ values and body temperature changes in mice. The computed values of respiratory rate (124 rpm), heart rate (387 bpm) and $SpO_{2}$ (88.9%) for 1.5% of sevoflurane were consistent with the ones documented for isoflurane. With this system, it was possible to verify the reported behavior for heart rate and respiratory rate evolution over time, with changes in the sevoflurane concentration. The heart rate is maintained in a well-defined range, and the respiratory rate increases with the decrease in the concentration of anesthetic agents and vice versa. The mice’s body temperature decreased over time, due to the lack of a heating system; however, this behavior proves the reliability of this sensor to detect hypothermia.

The non-invasive ECG has shown promising results, demonstrating the system’s capability to record all the ECG waves, allowing for direct measurement of the mice’s heart dynamics during image acquisition.

From the obtained data, the sensors used in the system are shown to be suitable to monitor the animal’s vital signs during the imaging procedures.

## Figures and Tables

**Table 1:** Respiratory rate (RR) values for the several concentrations of sevoflurane. P: value obtained with the piezoelectric sensor data; IR: value obtained with the IR LED data; Red: value obtained with the Red LED data.

Mouse	Sevoflurane (%)	RR±ΔRR (rpm)
1	1.5	P: 106 ± 2 IR: 105 ± 1 Red: 105 ± 1
	2	P: 124 ± 1 IR: 124 ± 1 Red: 124 ± 1
	4	P: 70 ± 1 IR: 71 ± 1 Red: 71 ± 1
2	2.5	P: 84 ± 1 IR: 84 ± 1 Red: 84 ± 1
	5	P: 39 ± 1 IR: 39 ± 1 Red: 39 ± 1

**Table 2:** Heart rate (HR) values for the several concentrations of sevoflurane. P: value obtained with the piezoelectric sensor data; IR: value obtained with the IR LED data; Red: value obtained with the Red LED data.

Mouse	Sevoflurane (%)	HR±ΔHR (bpm)
1	1.5	P: 387 ± 2 IR: 388 ± 3 Red: 388 ± 3
	2	P: 402 ± 6 IR: 392 ± 6 Red: 393 ± 7
	4	P: 374 ± 6 IR: 368 ± 5 Red: 369 ± 5
2	2.5	P: 407 ± 5 IR: 398 ± 3 Red: 405 ± 4
	5	P: 404 ± 3 IR: 401 ± 2 Red: 401 ± 3

**Table 3:** $SpO_{2}$ values for the several concentrations of sevoflurane.

Mouse	Sevoflurane (%)	${\mathrm{SpO}}_{2}\pm \Delta {\mathrm{SpO}}_{2}$ (%)
1	1.5	88.9 ± 0.6
	2	87.6 ± 0.6
	4	92.0 ± 0.3
2	2.5	89.5 ± 0.6
	5	88.7 ± 0.7

**Table 4:** Body temperature (BT) ranges for the two tested mice.

Mouse	BT±0.1 (${}^{{}^{\circ}}$C)
1	24.3–30.7
2	27.5–30.3

## Data Availability

Not applicable.
